# T-cadherin as a potential novel regulator of intestinal inflammation and fibrosis

**DOI:** 10.3389/fphys.2026.1946754

**Published:** 2026-09-29

**Authors:** Yan Wang, Ankita Prasad, Weiwei Liu, Jyotsna Chandra, Atif Zafar, Nayden G. Naydenov, Pranab K. Mukherjee, Andrei I. Ivanov, Florian Rieder

**Affiliations:** 1 Department of Inflammation and Immunity, Cleveland Clinic Research, Cleveland, OH, United States; 2 Program for Global Translational Inflammatory Bowel Disease Research, Cleveland Clinic, Cleveland, OH, United States; 3 Department of Gastroenterology, Hepatology and Nutrition, Digestive Disease Institute, Cleveland Clinic, Cleveland, OH, United States

**Keywords:** adiponectin, Crohn’s disease, inflammatory bowel disease, intestinal fibrosis, LDL, T-cadherin

## Abstract

T-cadherin (T-cad) is a non-canonical member of the cadherin superfamily with a distinguished structure of its glycosylphosphatidylinositol (GPI) anchor instead of transmembrane and cytoplasmic domains. This lends unique properties to and equips T-cad with dynamic mobility within the plasma membrane and extensive lateral interactions with signaling partners. Beyond its role as an adhesion molecule forming homophilic interactions, T-cad functions as a receptor for adiponectin and low-density lipoprotein (LDL), therefore playing an important role in regulating cellular processes such as proliferation, differentiation, and migration. These properties underscore its critical involvement in physiological and pathological processes, including central nervous system development, cancer progression, and cardiovascular disease. However, the role of T-cad in intestinal inflammation, injury, and fibrosis remains underexplored. This review highlights the structural and functional characteristics of T-cad, its binding partners, its expression and functions in various types of cells, tissues and organs, and its roles in health and disease. This review also speculates on its potential involvement in intestinal inflammation, particularly in stricturing Crohn’s disease.

## IBD and intestinal fibrogenesis

Inflammatory Bowel Disease (IBD) is a chronic inflammatory disease of the gastrointestinal tract that comprises of Crohn’s disease (CD) and ulcerative colitis (UC) (Xia et al., 1998). Chronic inflammation of the intestinal mucosa in IBD is clinically accompanied with episodes of abdominal pain, diarrhea, bloody stool, malabsorption and weight loss as major symptoms, and is linked to substantial tissue injury including mucosal ulceration (Xia et al., 1998). Prolonged responses to relapsing and remitting chronic inflammation and injury eventually leads to tissue fibrosis, leading to eventual stricture formation affected segments of the intestine (Rieder et al., 2024). About half of IBD patients develop stricture symptoms in their lifetime with partial or complete obstruction of intestinal lumen, eventually requiring surgical intervention (Xia et al., 1998).

## The role of cadherins in IBD

Cadherins are a superfamily of adhesion molecules that mediate Ca^2+^-dependent cell-cell adhesion in solid tissues with dense structural arrangement of cells and extracellular matrix that maintains a defined, non-fluid shape. This superfamily includes i) classical cadherins that are the major component of cell-cell adherens junctions (AJs) (e.g. E-cadherin, N-cadherin, VE-cadherin); ii) desmosomal cadherins (desmocolins and desmogleins); iii) protocadherins (μ-Protocadherin and CNR-cadherin); iv) other non-canonical cadherins including T-cadherin, 7TM-cadherin and FAT-family cadherin (Angst et al., 2001; Ivanov et al., 2001). Some classical cadherins are involved in the pathology of IBD. For example, the apical junctional complex (AJC) which consists of tight junctions, adherens junctions, and desmosomes, has crucial roles in maintaining intestinal epithelial barrier function and tissue homeostasis (Odenwald and Turner, 2017). As a component of the AJC, E-cadherin engagement is not only important for the formation of AJs, but also functions as the initial cue that recruits the key scaffolding proteins (including ZO-1, occluding and claudins) for tight junctions (TJs). TJs are the most apical component of the AJC and are directly responsible for the epithelial barrier. Without E-cadherin, these TJ proteins can no longer form a continuous, uninterrupted ring structure around the cell apex (Tunggal et al., 2005; Capaldo and Macara, 2007), leading to impaired epithelial barrier function, increased intestinal permeability and inflammation, and severe colitis (Zbar et al., 2004; Grill et al., 2015; Daulagala et al., 2019). Another member, P-cadherin, is expressed at low levels in normal intestinal cells, but upregulated in inflamed and injured mucosa in both CD and UC (Naydenov et al., 2022). Nayden et al. showed that P-cadherin may play a role in regulating epithelial cell migration and potentially contribute to the development of colitis-associated colon cancer (Naydenov et al., 2022).

Franze and colleagues (Franze et al., 2020) showed that the expression of cadherin-11(CDH-11) was increased in fibrotic stricturing tissue of both CD and UC, as compared with healthy controls. Knockdown of CDH-11 in fibroblasts isolated from CD patients inhibited RhoA/ROCK/TGF-β signaling and suppressed collagen synthesis. In addition, CDH-11 knockout (KO) protected the mice against TNBS (trinitrobenzene-sulphonic acid)-induced colitis-associated colonic fibrosis. A recent publication from our group using the first full thickness single-cell RNA sequencing atlas confirms that CDH-11 expression is upregulated in IBD tissues and myofibroblasts, and emerges as a dominant cell surface molecule in CD strictures. Modulation of CDH-11 expression affects the extracellular matrix (ECM) production by myofibroblasts. In addition, functional ablation of CDH-11 alleviates intestinal experimental fibrosis (Mukherjee et al., 2023).

As the major component of AJs and the key regulator of endothelial barrier function, VE-cadherin is one of the primary targets in vascular permeability disruption induced by inflammation (Dejana et al., 2008; Britzen-Laurent et al., 2023). Langer and colleagues (Langer et al., 2019) reported that in both dextran sulfate sodium (DSS)-induced murine colitis model and human IBD patients, membrane VE-cadherin expression was significantly reduced in blood vessels in inflamed areas as compared to uninvolved intestinal tissues. In addition, as a major cytokine in IBD pathogenesis, IFN-γ drove the pathology of IBD mainly by causing the disruption of VE-cadherin-mediated vascular barrier function. Moreover, endothelial-specific knockout of IFN-γ receptor 2 ameliorated the DSS-induced colitis in mice. All these findings suggest that VE-cadherin has an important role in the development of IBD pathology.

## Introduction of T-cadherin

T-cadherin (derived from “T” as “truncated”, abbreviated as T-cad), with the official gene name as *CDH13*, also known as H-cadherin or cadherin-13, is a non-canonical member of the cadherin superfamily. It has the typical five calcium-binding extracellular domains for cadherins, but is devoid of transmembrane and cytoplasmic domains. Therefore, it does not have strong adhesive capability like other canonical cadherins. Instead, T-cad is attached to the plasma membrane via a cosyGPI anchor, which renders T-cad very dynamic and capable of fast movements within the plasma membrane and lateral interactions with its signaling partners (George and Beeching, 2006). In the plasma membrane, T-cad is present in caveolin-rich lipid rafts due to the GPI anchor effect (Doyle et al., 1998; Philippova et al., 2003). In contrast to classical cadherins that are typically localized at intercellular AJs, T-cad shows a global and diffuse distribution across the cell body in the resting monolayer of cells such as endothelial cells, smooth muscle cells and tumor cells such as bladder transitional cell carcinoma (Lin et al., 2013). T-cad is also found concentrated in the leading edges of migrating vascular cells (Philippova et al., 2003).

## Gene organization and regulation of T-cadherin

The T-cad gene is evolutionarily conserved and displays strong homology among different species (Philippova et al., 2009). The human T-cad gene locates on chromosome 16q24 (Lee, 1996) and mouse T-cad gene on chromosome 8 (Leduc et al., 2011). The human T-cad gene consists of 1,169,627 base pairs, containing 14 exons (the first and fourteenth exons untranslated) (Redies et al., 2005), producing a transcript of 3711 bps, which translates into a 713 amino acid preprotein (Ranscht and Dours-Zimmermann, 1991). Like other cadherins such as E-cadherin, the T-cad promoter does not have a TATA box, which has an important role in transcription initiation and regulation. However, there are regulatory elements and potential binding sites for estrogen receptor, glucocorticoid receptor and progesterone receptor present in the 5’-flanking region (Bromhead et al., 2006), and estradiol, progesterone, Epidermal Growth Factor (EGF) and dexamethasone are involved in transcriptional and posttranscriptional regulation of T-cad in human osteosarcoma cells (Bromhead et al., 2006). In addition, several putative regulatory elements were proposed to reduce T-cad transcriptional activity including an androgen response element (ARE) located within the T-cad promoter showing a suppressive effect on T-cad expression (Wang et al., 2007), and an aryl hydrocarbon response (AHR) element negatively regulating T-cad expression in rat vascular smooth muscle cells when being treated with the AHR agonists benzo(*a*)pyrene (BaP) or 7,12-dimethylbenzanthracene (DMBA) or 2,3,7,8-tetrachlorodibenzo-*p*-dioxin (TCDD) (Niermann et al., 2003). Other known mechanisms that negatively regulate T-cad expression include: growth factors such as Platelet Derived Growth Factor-BB (PDGF-BB), Epidermal Growth Factor (EGF), and Insulin Growth Factor (IGF) can downregulate T-cad expression in rat vascular smooth muscle cells (Kuzmenko et al., 1998); transcriptional regulator Zeb1 binds to the T-cad promoter and represses its activity in E-box-like sequence-dependent fashion (Adachi et al., 2009); DNA methyltransferase 3b (Dnmt 3b) can suppress T-cad expression during nerve growth factor-induced PC12 cell differentiation, suggesting the association of histone deacetylase and epigenetic regulation of the T-cad promoter (Bai et al., 2006). On the other hand, T-cad expression can be induced by oxidative stress in endothelial cells (Joshi et al., 2005). So far there are few reports about how the expression of T-cad changes in inflammation and especially chronic inflammation. Therefore, how proinflammatory cytokines regulates T-cad expression remains to be elucidated.

## Protein structure of T-cadherin

Canonical cadherins are transmembrane glycoproteins consisting of an amino-terminal extracellular domain with 5 subdomains (EC1-EC5), a transmembrane domain, and a C-terminal cytoplasmic domain. The extracellular domain mediates Ca2+-dependent homophilic interactions by forming dimers via N-terminal EC1 domain interactions and “strand swapping” between partner molecules of the cadherins on the surface of the neighboring cells (Harrison et al., 2010). In addition, cis interactions of the EC domains of different cadherins causes clustering of cadherins. The cytoplasmic domains of canonical cadherins bind to members of the catenin protein family including β-catenin and p120 catenin, or bind to F-actin. These binding activities stabilize adhesive clusters and promote the maturation of AJs, and connect AJs to the actin cytoskeleton system functionally (Mege and Ishiyama, 2017). As a non-canonical member of the cadherin superfamily, the T-cad amino acid sequence is well conserved in vertebrates. T-cad preprotein has 713 amino acids and consists of a pro-peptide, five cadherin domains and a GPI anchor attached to the carboxyterminal end (Ranscht and Dours-Zimmermann, 1991). The EC1 domain of T-cad exhibits 30% protein similarity with that of other classical cadherins (Dames et al., 2008). However, T-cad EC1 does not have amino acids important for the adhesive functions of classical cadherins, including tryptophan at position 2 that forms the classical cadherin strand exchange dimers, and the His-Ala-Val motif which is conserved between canonical cadherins and required for strong homophilic adhesion between type I cadherins (Ranscht and Dours-Zimmermann, 1991). Instead, T-cad has an isoleucine residue at position 2 and only forms relatively small adhesive pockets on the EC1 domain. Therefore, T-cad can only form x-dimer binding intermediates via a non-swapped interface near EC1-EC2 Ca2+-binding sites (Ciatto et al., 2010). Without an intermembrane domain and a cytoplasmic tail, T-cad possesses a hydrophobic sequence of 19 amino acids on the C-terminus that functions as a signal for GPI-anchoring attachment. It is attached to the plasma membrane via a glycosyl phosphatidylinositol moiety.

Western blotting of T-cad identified multiple bands ranging between 45 kDa and 130 kDa, with two major bands: one is 130 kDa as the pro-protein, one is 105 kDa as the mature protein (Stambolsky et al., 1999). It is believed that different T-cad precursors or splice variants exist in different type of tissues (Ranscht and Dours-Zimmermann, 1991). T-cad is also highly glycosylated and contains eight potential N-glycosylation sites (Joshi et al., 2009) and a single O-mannosylation site (Winterhalter et al., 2013). The functional roles of T-cad variants and protein isoforms remain to be elucidated. One example is that the expression of specific variants of T-cad show correlation with adult attention deficit/hyperactivity disorder (ADHD) (Mavroconstanti et al., 2013), diabetic nephropathy (Nicolas et al., 2017b) and fatty liver in particular populations (Nicolas et al., 2017a).

## Biologic ligands binding to T-cadherin

Several biological ligands including low-density lipoprotein (LDL) and adiponectin have been reported to signal through T-cad. LDL mainly functions as the carrier of cholesterol to peripheral tissues and binds to both the 130 kDa precursor and the 105 kDa mature protein of T-cad (Bochkov et al., 1996). Interestingly, it was shown that the GPI anchor is required for the binding between T-cad and LDL (Niermann et al., 2000). However, the underlying molecular and structural characteristics of LDL binding to T-cad remain unclear. It was reported that the binding of LDL to T-cad increased intracellular calcium concentration in human umbilical vein endothelial cell lines and human embryonic kidney cells (Kipmen-Korgun et al., 2005; Rubina et al., 2005), and resulted in an activation of ERK1/2 and NF-kB, and increased cell proliferation (Kipmen-Korgun et al., 2005). However, the functional relevance of LDL binding to T-cad has not been fully appreciated, and the tissue/cell context-specific binding between LDL and T-cad remains to be further elucidated.

Adiponectin is the most abundant adipokine secreted mainly by white adipose tissue (WAT) adipocytes. Also referred to as Acrp30, GBP-28, apM1, and AdipoQ, adiponectin is encoded by ADIPOQ gene in humans (Maeda et al., 1996). Adiponectin is composed of 247 amino acids and four domains: an N-terminal collagen-like domain, a signal sequence for the extracellular secretion, a hypervariable region that varies among different species, and a C-terminal globular domain (Shapiro and Scherer, 1998). Adiponectin proteins undergo extensive posttranslational modification by glycosylation and hydroxylation, which renders the proteins into multimerization to form trimers, hexamers, and high-molecular-weight (HMW) oligomers. Adiponectin can bind to three receptors, AdipoR1 and AdipoR2 (Nguyen, 2020), and T-cad. However, only the hexamers and the HMW isoform (as the main form) bind to T-cad (Hug et al., 2004). Adiponectin binding requires the EC1-EC2 domains of T-cad, which overlap with the region important for T-cad-mediated homophilic interaction on the contacting cells (Ciatto et al., 2010; Fukuda et al., 2017). Binding of HMW adiponectin induces strong clustering and internalization of T-cadherin without affecting calcium signaling. The main biological functions of adiponectin include promoting fatty acid synthesis but inhibiting gluconeogenesis in the liver, and increasing glucose uptake in skeletal muscle. Therefore, adiponectin has a well-recognized role as an insulin sensitizer and anti-diabetic (Choi et al., 2020). However, its role in regulating inflammation turns out to be complicated and dependent upon different disease models and types of tissues and organs (Choi et al., 2020). It was shown that T-cad deficiency impaired adiponectin-mediated revascularization in mice (Parker-Duffen et al., 2013), and abrogated cellular migration and proliferation enhanced by adiponectin. T-cad is also involved in muscle regeneration driven by adiponectin signaling (Tanaka et al., 2019).

## Expression and function of T-cadherin in various types of tissues

T-cad expression was found in various types of tissues including the central nervous system (CNS), cardiovascular system (arteries, atrial appendage and left ventricle), adipose tissue, skeletal muscle and epithelium such as lung, kidney, skin, mammary, uterus, vagina, prostate, colon and intestine. Low levels of T-cad were also detected in tissues such as pancreas and liver (Consortium TG-TEG, 2020).

Nervous system: In the human adult CNS, strong T-cad expression is detected in the cerebral cortex, medulla, thalamus, and midbrain. Little or no expression of T-cad was detected in the spinal cord or in developing brains (Ranscht and Bronner-Fraser, 1991; Takeuchi et al., 2000). T-cad expression is found particularly in neurons in the substantia nigra pars compacta and in the ventral tegmental area that are involved in locomotion control, reward memory and cognitive modulatory functions. T-cad has an important role in guiding neuron growth during development and modulating specific synaptic contacts and neural circuit formations in the brain (Fredette and Ranscht, 1994; Hayano et al., 2014). By forming low-adhesive homophilic interactions, T-cad acts as a boundary marker and functions as a negative guidance cue for neuron growth and circuit wiring (Forero et al., 2017). Several genome-wide association studies (GWAS) identified T-cad genetic variants as risk factors in association with several neuronal developmental disorders including ADHD, drug and alcohol abuse, and mental health diseases such as schizophrenia, autism, bipolar behavior and personality traits (Treutlein et al., 2009; Arias-Vasquez et al., 2011; Mavroconstanti et al., 2013). T-cad is also found downregulated in gliomas and overexpressing T-cad in C6 glioma cells induced aneuploidy and G2 phase growth arrest, suggesting T-cad may have a role in neuronal oncogenesis (Huang et al., 2003).

Skeletal muscles: T-cad is detected in skeletal muscle and co-localizes with adiponectin here. In a cardiotoxin-induced muscle injury mouse model, it was shown that adiponectin accumulates in regenerating muscle fibers of tibialis anterior muscles (Tanaka et al., 2019). Overexpression of adiponectin *in vivo* alleviated muscle injury and improved muscle regeneration in adiponectin null mice but not in T-cad null mice. Knockdown of T-cad in differentiating C2C12 myotubes reduced adiponectin accumulation and adiponectin-mediated exosome production, suggesting that T-cad mediates adiponectin’s function in promoting muscle regeneration (Tanaka et al., 2019).

Lung and Kidney: T-cad is mainly detected in bronchioloalveolar type II cells in the lung (Takeuchi et al., 2001), and its expression is downregulated in this cell type in patients with secondary pulmonary alveolar proteinosis associated with hematolymphoid malignancies. Kasahara et al. (2013) reported that T-cad is required for adiponectin-mediated suppression of ozone-induced pulmonary inflammation. T-cad appears to have a paradoxical role in the development of lung fibrosis, dependent upon the stage, the specific disease trigger and cell-to-cell signaling mechanisms. The expression of T-cad is significantly reduced in patients with idiopathic pulmonary fibrosis (Nemeth et al., 2024). In lung fibroblasts cultured *in vitro*, T-cad binds to adiponectin and activates p38MAPKγ pathway, and suppresses the activation of scar-forming lung fibroblasts and slows down ECM production and stiffness development (Nemeth et al., 2024). On the other hand, Semina et al. reported a significant reduction in T-cad expression in post-mortem lung tissues from COVID-19 patients, and this decreased T-cad expression correlated with increased expression of VE-cadherin. Furthermore, T-cad knockout mitigates bleomycin induced pulmonary fibrosis (Semina et al., 2025). In renal tissue, Tsugawa-Shimizu and colleagues (Tsugawa-Shimizu et al., 2021) reported that the expression of both T-cad and its ligand, adiponectin, was detected in the platelet derived growth factor receptor β (PDGFRβ)-positive pericytes surrounding peritubular capillaries, and the existence of T-cad was important in maintaining the renal capillary network integrity and preventing the pericyte loss during ischemia-reperfusion injury in their mouse model. T-cad gene expression polymorphisms were found to be associated with type-I diabetes associated nephropathy (Nicolas et al., 2017b). In their subtotal nephrectomy-induced chronic kidney model, Ohashi et al. reported that, urinary albumin excretion, glomerular hypertrophy, and tubulointerstitial fibrosis were much worse in Adiponectin-KO mice compared with WT ones, and overexpression of Adiponectin resulted in amelioration of all the symptoms. However, whether this effect is T-cad-dependent or not was not addressed (Ohashi et al., 2007).

Cardiovascular system: T-cad is widely found in the human cardiovascular system. High expression of T-cad is detected in aortic endothelial cells, smooth muscle cells, pericytes, and cardiomyocytes (Ivanov et al., 2001). In wild-type mice, T-cad appears to co-localize with adiponectin, and the expression levels of the two appear to interconnect with each other in the cardiovascular system (Denzel et al., 2010). For instance, in T-cad KO mice, adiponectin is barely detectable locally in cardiac tissue, but its levels were significantly increased in the serum of the same mice (Denzel et al., 2010). T-cad levels were also reduced in adiponectin KO mice (Matsuda et al., 2015). T-cad appears to have important functions in binding and sequestering circulating adiponectin in cardiovascular tissues, and is critical for adiponectin-mediated cardioprotection (Clark et al., 2017). Kondo Y et al. showed that administration of soluble T-cad significantly attenuated the heart failure and cardiac fibrosis in the mice induced by the transverse aortic constriction (TAC) surgery, and suppressed the upregulated Acta2 gene expression in NIH-3T3 fibroblasts induced by TGF-β signaling, suggesting that T-cad has a crucial role in regulating cardiac fibrosis (Kondo et al., 2025).

Endothelial cells: T-cad has a complex role in regulating endothelial barrier function. A baseline level of T-cad is required to maintain endothelial barrier integrity, and knockdown of T-cad expression modestly reduces transendothelial resistance (Andreeva et al., 2010). Conversely, pathological upregulation of T-cad, which is frequently observed in atherosclerosis and post-angioplasty restenosis (Qin et al., 2003), disrupts endothelial barrier by inducing the phosphorylation of VE-cadherin at Y731 residue, which leads to the clathrin-dependent endocytosis and of VE-cadherin and its subsequent lysosomal degradation, resulting in the breakdown of intercellular junctions and vascular leakage (Semina et al., 2014). In addition, T-cad overexpression induces Rho GTPases activation which drives the formation of actin stress fibers that physically pull endothelial cells apart (Semina et al., 2014). In spite of its paradoxical role in regulating endothelial barrier function, T-cad overexpression protects endothelial cells against stress-induced apoptosis via the activation of PI3K/Akt/mTOR signaling pathway and increases the expression and nuclear accumulation of active β-catenin (Joshi et al., 2005). The association of T-cad with both glucose-related protein GRP78 and integrin β3 on the cell surface is essential in mediating the cell survival function (Philippova et al., 2008). Integrin-linked kinase (ILK), a member of the Raf-like kinase subfamily that is involved in integrin-mediated signal transduction, has been shown to be associated with T-cad in endothelial cells and inhibition of ILK expression abrogated the role of T-cad in promoting PI3K/Akt activation and active β-catenin accumulation and cell survival (Joshi et al., 2007). T-cad also plays an important role in protecting endothelial cells against endoplasmic reticulum (ER)-induced stress and apoptosis (Kyriakakis et al., 2010). Obata and colleagues showed that T-cad/adiponectin enhances exosome biogenesis and ceramide efflux, protecting the endothelium from oxidative stress and blocks toxic intracellular ceramide accumulation (Obata et al., 2018). In addition to increased resistance to apoptosis, T-cad-mediated PI3K/Akt pathway activation also increases phosphorylation of the key downstream effectors including GSK-3β, S6RP, P70S6K, and IRS-1 in endothelial cells, which have critical roles during the development of insulin resistance (Philippova et al., 2012; Frismantiene et al., 2014).

T-cad also has an important role in regulating angiogenesis. Parker-Duffen et al. (2013) showed that in a hind limb ischemia model where blood flow is disrupted in one limb of mouse and recovery is monitored over a period of 28 days, the blood flow recovery was impaired in both T-cad-deficient mice and adiponectin-deficient mice, as compared with the WT controls. In addition, administration of exogenous adiponectin restored the impaired revascularization only in adiponectin-deficient mice, but not in T-cad-deficient ones. The authors further showed that knockdown of T-cad by RNA interference in the cell culture model abolished adiponectin’s role in promoting cell migration and proliferation of endothelial cells (Parker-Duffen et al., 2013). Lastly, the expression levels of T-cad on endothelial cells in human aorta or coronary arteries were found negatively correlated with the severity of atherosclerosis (Kostopoulos et al., 2014). In addition, either adiponectin KO or T-cad KO exacerbated the atherosclerotic pathology in apolipoprotein E-KO mice (Fujishima et al., 2017). Since the endothelium is one of the most important regulators of vascular homeostasis, T-cad could potentially have an important role in many vascular pathologies by regulating endothelial functions in response to acute or chronic stress.

Vascular smooth muscle cells (VSMCs): In contrast to its lower expression in endothelial cells in atherosclerosis-prone porcine coronary arteries (Qin et al., 2003), T-cad expression was found increased in smooth muscle cells (SMCs) of atherosclerosis (Ivanov et al., 2001). Frismantiene and colleagues showed that overexpression of T-cad in VSMC induced constitutive activation of Akt/GSK3β axis and its downstream signaling (S6RPser235/236 and IRS-1ser636/639). In addition, overexpression of T-cad promoted collagen fibril reorganization and the contractility of VSMCs. Conversely, T-cad knockdown yielded the opposite effect (Frismantiene et al., 2014). The same group further showed that overexpression of T-cad promoted VSMCs migration and proliferation but downregulated the expression of α-SMA and other SMC differentiation markers, and impaired stress fiber formation in VSMCs. Ectopic expression of constitutively active GSK-3beta restored the expression of differentiation markers and blocked hyperproliferation in T-cad+-VSMCs (Frismantiene et al., 2016). Ectopic expression of T-cad in VSMCs also enhanced autophagy characterized by increased autophagic flux, LC3-II abundance and autophagosome formation. This autophagy induction was dependent on MEK1/2, ERK1/2 activation and provided a survival advantage upon prolonged serum starvation. Conversely, T-cad deficiency in SMCs diminished autophagy responsiveness and compromised survival in response to serum starvation (Kyriakakis et al., 2017).

Pericytes: Pericytes are mesenchymal cells lining capillaries and small blood vessels, and control the function of endothelial cells (Gerhardt and Betsholtz, 2003). Pericytes can differentiate into fibroblast-like cells and contribute to inflammation-associated fibrosis (Sundberg et al., 1996). T-cad was found expressed on pericytes isolated from either mouse heart and brain, or human adipose tissue (Dasen et al., 2023). Using lentivirus-mediated T-cadherin overexpression or silencing in cultured human pericytes, Dasen and colleagues demonstrated that T-cad had a crucial role in regulating pericyte proliferation, migration, invasion, and interactions with endothelial cells during angiogenesis. These regulatory effects were associated with the reorganization of the cytoskeleton and mediated by Akt/GSK3β and ROCK intracellular signaling pathways (Dasen et al., 2023).

Cancer cells: The gene location of T-cad is prone to oncogenic modifications. Locus deletion or methylation of the promoter region of the T-cad gene is commonly found in cancers. Gene expression of T-cad is downregulated or silenced in various types of cancers including breast (Lee, 1996; Hebbard et al., 2008), lung (Sato et al., 1998), digestive gastric and colorectal (Mori et al., 1999; Toyooka et al., 2002; Hibi et al., 2004), ovarian (Takeuchi and Ohtsuki, 2001), prostate (Dasen et al., 2017) and skin (Takeuchi et al., 2002). For example, Lee showed that T-cad expression was downregulated in breast cancer and induced expression of T-cad in tumor cells significantly diminished cell growth and resulted in an evident change in cellular morphology in the matrigel outgrowth assay (Lee, 1996). Therefore, T-cad is regarded as a tumor suppressor in many types of cancers. On the other hand, Hebbard and collagues (Hebbard et al., 2008) reported that T-cad deficiency inhibited mammary tumor angiogenesis and reduces tumor growth. In addition, adiponectin was found associated with T-cad-positive vasculature in both normal and malignant mammary glands but missing in the T-cad null tissue, suggesting that the association between T-cad and adiponectin in vasculature may mediate the interaction between tumor cells and the stromal microenvironment in breast cancer. In addition, T-cad is not detected on hepatocytes in normal liver but its expression is upregulated on tumor cells of hepatocellular carcinoma tissues (Riou et al., 2006), suggesting that *de novo* expression of T-cad may drive pathogenesis of disease. It was reported that hypermethylation in the promoter region of T-cad and downregulation of T-cad expression occurred in human colon carcinoma cells (Ren and Huo, 2012). Furthermore, a significant association between T-cad methylation and poorly differentiated colorectal cancers has been reported (Hibi et al., 2004), suggesting that inactivation of T-cad gene expression could contribute to the formation of scattered carcinoma cells in colorectal cancers. All the discrepant observations suggest a cell-context-dependent and ligand-dependent role of T-cad in tumorigenesis.

In summary, T-cad is detected in various types of cells and tissues. By forming a homodimer with another T-cad molecule from adjacent cells, or by binding to ligands including LDL or adiponectin, T-cad has overarching functions in regulating various cellular processes. However, T-cad’s functions diverge largely depending upon which partner it binds to, and what downstream signaling pathways are activated in different cell and tissue types (Rubina et al., 2021). The binding partners and intracellular signaling pathways of T-cad is summarized in **Figure 1**.

**Figure 1:**
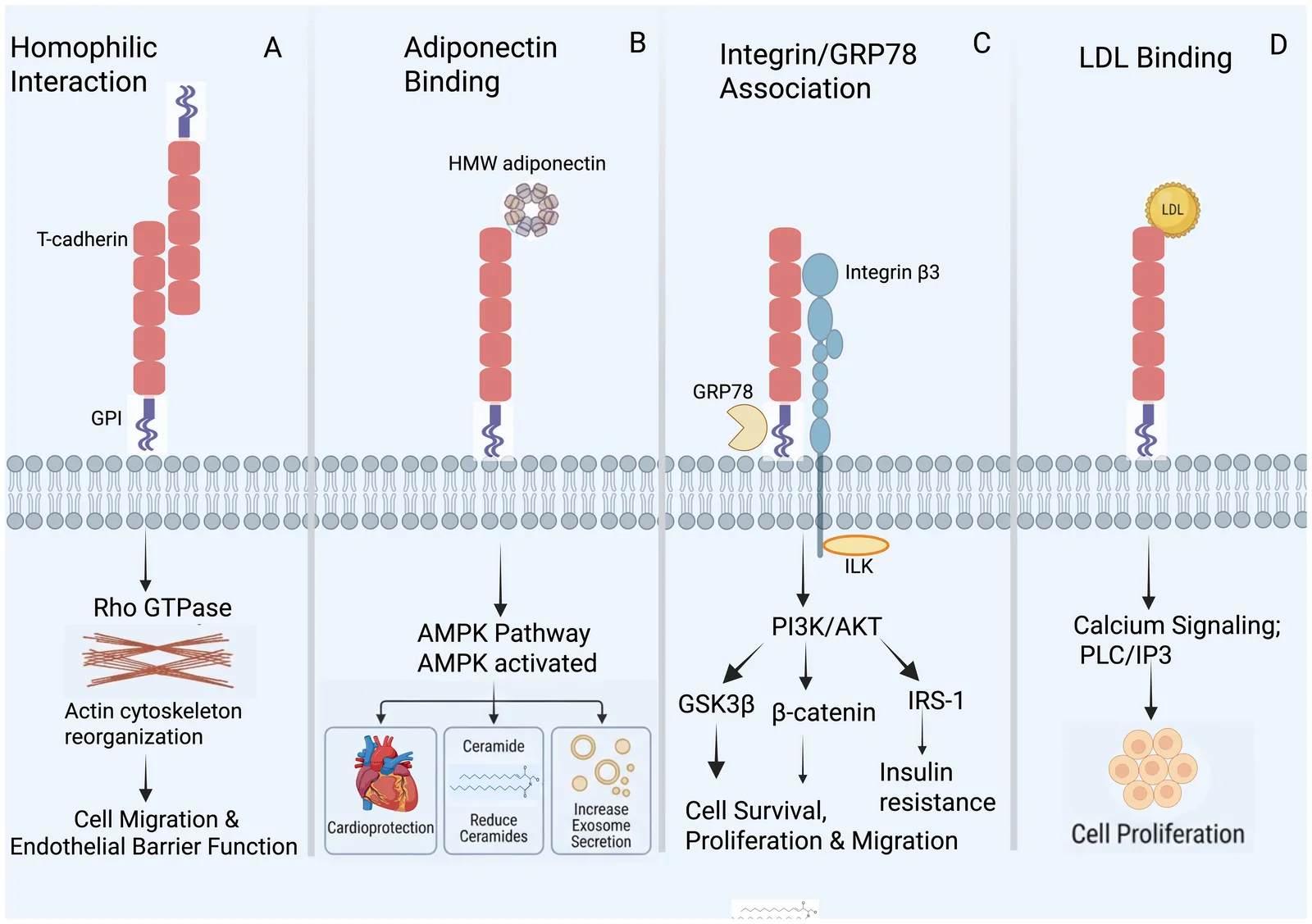
T-cadherin binding partners, intracellular signaling pathways and cellular functions. **(A)** T-cadherin can form homophilic interaction between EC1-EC3 domains on the neighboring cells leading to Rho GTPase activation which regulates cell migration and endothelial barrier function. **(B)** The binding of high molecular weight (HWM) adiponectin to T-cadherin induces the activation of AMPK pathway in endothelial cells and cardiomyocytes, resulting in increased exosome secretion and ceramides efflux, which have crucial roles in cardioprotection. **(C)** Association of T-cadherin with Glucose-Related Protein 78 (GRP78) and integrin β3 on the cell surface induces ILK activation, which leads to PI3K/AKT activation and the subsequent activation of downstream signaling targets including GSK3β, β-catenin, and Insulin Receptor Substrate-1 (IRS-1). Activation of GSK3β and β-catenin affects cell survival and proliferation; activation of IRS-1 is involved in insulin resistance. **(D)** The binding of LDL to T-cadherin triggers an increase in intracellular calcium concentration and the activation of Phospholipase C (PLC)/IP3 pathway which affects cell proliferation. Created in BioRender. Wang, Y. (2026) https://BioRender.com/yg8ryd6.

## The role of T-cad and its ligands in intestinal inflammation and fibrosis

Despite robust evidence on T-cad’s function in various types of cells in the cardiovascular system, central nervous system and cancer, surprisingly there is no report on the association of T-cad with IBD, except for a study that reported a promoter hypermethylation of the *CDH13* gene in severe UC patients (Tahara et al., 2014). As one of the main and most devastating complications of IBD, intestinal fibrogenesis is characterized by excessive ECM protein deposition. ECM is mainly produced by activated myofibroblasts, that are derived from fibroblasts and represent the major effector cell that mediates tissue fibrosis. However, various types of cells may transdifferentiate into activated myofibroblasts that produce ECM during the development of intestinal fibrosis (Rieder et al., 2024). These cells derive not only from resident fibroblasts or smooth muscle cells, but also from epithelial cells via epithelial-mesenchymal transition (EMT). Endothelial cells can also differentiate into myofibroblasts via endothelial-mesenchymal transition (EndoMT) (Rieder et al., 2011). Stellate cells and pericytes from the capillaries and small blood vessels, and stem cells from bone marrow are also sources for activated myofibroblasts (Rieder and Fiocchi, 2009). In addition to the excessive deposition of ECM in the gut, a thickening of the muscularis propria also occurs (Veisman et al., 2024). In fact, thickening of the muscularis propria is considered the strongest contributor to luminal narrowing in patients with obstructive symptoms. However, mechanisms of muscularis propria muscle cell proliferation are still poorly understood (Rieder et al., 2013). A recent study from our group demonstrated that long-chain free fatty acid released by creeping fat induced a specific strong proliferation of human intestinal muscle cells, suggesting creeping fat as a novel contributor to stricture formation in Crohn’s disease (Liu et al., 2025). Angiogenesis also has an important function in the pathogenesis of IBD by supplying nutrients and oxygen to the injured region perpetuating inflammation, and chemotaxis of inflammatory cells, and also drainage of lymphatics from the tissue (Koutroubakis et al., 2006; Alkim et al., 2015). In IBD, physiological angiogenesis turns into a pathological process at the early stages of disease. Although there is no report connecting T-cad to IBD, its important role in regulating various cell types including endothelial cells, VSMC and pericytes as discussed above suggests its potential involvement in the pathology of IBD. The comparison between T-cad and other members of the cadherin family in structure and function, especially their role in the pathology of IBD is summarized in **Table 1**.

**Table 1:** Comparison of different types of cadherins in their structure, expression, function, and role in IBD.

Cadherin type	Structure	Primary cell/tissue type	Function	Alteration in IBD	Primary pathological role & mechanism
E-cadherin (Cadherin-1)	Type I Classical: 5 extracellular (EC) domains with calcium-binding motifs; single transmembrane domain; highly conserved cytoplasmic tail linking to actin via catenins.	Epithelial cells (e.g. skin, gastrointestinal lining)	Establishes cell-to-cell adhesion, maintains epithelial structural integrity, dictates cell polarity, and suppresses tumor cell invasion.	Downregulated or structurally disrupted in active lesions (Zbar et al., 2004; Grill et al., 2015; Daulagala et al., 2019)	Loss weakens the epithelial adherens, impairs barrier function, triggering a leaky gut and increased inflammation (Zbar et al., 2004; Grill et al., 2015; Daulagala et al., 2019).
P-cadherin (Cadherin-3)	Type I Classical: Similar to E-cadherin with 5 EC domains and conserved catenin-binding cytoplasmic region.	Basal layer of epidermis, placenta, breast myoepithelial cells.	Promotes tissue cohesion, drives segregation of basal cells, and regulates localized cell proliferation.	Upregulated in inflamed/injured mucosa (Naydenov et al., 2022).	It acts as a negative regulator of epithelial cell migration and its upregulation *slows down* mucosal wound healing (Naydenov et al., 2022).
Cadherin-11 (OB-cadherin)	Type II Classical: 5 EC domains but lacks the specific type-I conserved HAV sequence motif; standard transmembrane and catenin-binding tail.	Mesenchymal cells, osteoblasts, fibroblasts, and synovial linings	Mediates mesenchymal tissue remodeling, osteoblast differentiation, joint synovial lining development, and neural crest cell migration.	Significantly increased in both CD and UC (Franze et al., 2020; Mukherjee et al., 2023).	Expressed on intestinal fibroblasts. Pro-inflammatory cytokines (TNF-α, IL-6, IL-1β) stimulate its expression, which activates RhoA/ROCK/TGF-β signaling, heavily driving excessive collagen synthesis and tissue scarring (Franze et al., 2020, Mukherjee et al., 2023).
VE-cadherin (Cadherin-5)	Type II Classical: 5 EC domains with distinct structural packing variations; single transmembrane domain connecting to vascular signaling pathways.	Vascular endothelial cells (blood and lymphatic vessel linings).	Maintains endothelial barrier function, controls vascular permeability, regulates angiogenesis, and manages leukocyte extravasation.	Downregulated or structurally disrupted by localized cytokines such as Interferon-gamma (IFN-γ) (Langer et al., 2019).	Vascular Leakage & Leuko-infiltration: High levels of IFN-γ in the inflamed gut cause a targeted breakdown of VE-cadherin at endothelial borders. This collapses the *gut-vascular barrier*, causing profound vessel permeability and a massive influx of inflammatory cells into the tissue (Langer et al., 2019).
T-cadherin (Cadherin-13/H-cadherin)	Atypical Non-classical: Contains 5 EC domains but completely lacks a transmembrane and cytoplasmic domain; anchored via a GPI-anchor.	Cardiomyocytes, vascular smooth muscle cells, endothelial cells, neurons.	Acts as a surface receptor rather than a direct mechanical linker; binds adiponectin and LDL, coordinating cardio-protection and guiding signals.	Hypermethylation of *CDH13* gene promoter in severe UC patients (Tahara et al., 2014)	Functions with its ligand adiponectin and regulates the creeping fat formation and the function of other types of cells such as SMC in IBD?

The role of the T-cad ligand adiponectin has been investigated in IBD. There was no difference in serum adiponectin levels between UC and CD patients compared to controls. However, significantly higher serum adiponectin levels were observed in UC compared to CD patients (Surdea-Blaga et al., 2023). Studies characterizing adiponectin locally in the gut have been focused on adipose tissues. Rodrigues and colleagues reported that adiponectin expression in mesenteric adipose tissue was lower in CD patients as compared to that of non-CD controls (Rodrigues et al., 2012). Yamamoto et al. (2005) showed that the expression of adiponectin was upregulated in creeping fat of CD patients as compared to non-creeping fat of CD patients and fat from ulcerative colitis patients, suggesting the possible involvement of adiponectin in regulating the intestinal inflammation associated with CD. Creeping fat is a form of hyperplasia of the mesenteric adipocytes leading to wrapping of the fat around the intestine (Kredel and Siegmund, 2014). This is highly relevant as creeping fat is linked with stricturing CD (Liu et al., 2025). As a classic anti-inflammatory adipokine, adiponectin’s upregulation in creeping fat most likely represents one of the body’s primary defense mechanisms to contain transmural inflammation. As the receptor for adiponectin, T-cad appears to play a negative role in regulating adipogenesis under homeostatic conditions. Sysoeva et al. showed that mesenchymal stem cells (MSCs) isolated from T-cad-deficient mice had an enhanced adipogenic potential and underwent spontaneous differentiation into adipocytes with the formation of large lipid droplets (Sysoeva et al., 2024). In addition, although both low molecular weight (LMW) and high molecular weight (HMW) adiponectin have suppressive effects on adipogenesis, only HMW adiponectin’s effect was impacted by T-cad deficiency (Sysoeva et al., 2024). A more recent study also reported that T-cad overexpression sustained 3T3-L1 fibroblasts in a proliferative and undifferentiated preadipocyte state, whereas T-cadherin depletion accelerated the adipogenic differentiation and enhanced lipid accumulation by activating PI3K-AKT pathway (Klimovich et al., 2025). All the findings suggest that the T-cad-adiponectin axis functions as a key modulator of adipose tissue plasticity. However, their role in regulating creeping fat in CD patients remains to be further elucidated. As for the function of adiponectin in colon epithelial cells, Ogunwobi and Beales (2006) showed that globular adiponectin enhanced cell proliferation and cytokine secretion and protected the cells against apoptosis using the model of human HT-29 colonic cancer epithelial cells. However, divergent findings on adiponectin’s role in the pathogenesis of IBD were generated from *in vivo* mouse models: Nishihara and colleagues (Nishihara et al., 2006) showed that adiponectin protects mice against acute colitis induced by dextran sulfate sodium (DSS). Consistent with the above findings, another study showed that adiponectin-deficient mice developed more severe colonic inflammation, increased apoptosis of intestinal epithelial cells, and exhibited severe colonic crypt disruption upon acute DSS treatment. This was accompanied with extensive B cell infiltration, and increased levels of pro-inflammatory cytokines such as IL-1β, IL-4, and IL-6 (Obeid et al., 2017). In contrast, Fayad and colleauges (Fayad et al., 2007) indicated that systemic adiponectin administration via intraperitoneal injection induced the production of pro-inflammatory cytokines such as IL-6 and MIP-2 in acute DSS-treated mice, and genetic depletion of adiponectin protected mice against DSS-induced colitis. In a porcine AdipoR1 transgenic mouse model, DSS-induced colitis showed a higher severity, and adiponectin treatment increased the production of proinflammatory cytokines in human colon epithelial cell lines (Peng et al., 2018). Discrepancies between these studies likely arise from variations in experimental protocols, reagent selection, mouse lineages, and facility-specific microbiomes. For example, two groups using different lineages of adiponectin-KO mice demonstrated conflicting phenotypes in the development of diet-induced insulin resistance (Maeda et al., 2002; Ma et al., 2002). In addition to AdipoR1, whether T-cad has a role in mediating adiponectin’s function in IBD as its receptor remains to be elucidated. The studies described as above are summarized in **Table 2**.

**Table 2:** Published studies characterizing the role of adiponectin in IBD.

Key findings	References
Serum level of adiponectin is significantly higher in UC patients than in CD patients	Surdea-Blaga et al. (2023)
Adiponectin expression is significantly lower in mesenteric adipose tissue of CD patients than in non-CD patients	Rodrigues et al. (2012)
Adiponectin expression is significantly higher in creeping fat of CD patents as compared to non-creeping fat of CD patients or fat from UC patients	Yamamoto et al. (2005)
Globular adiponectin enhances cell proliferation & cytokine secretion, and protects against apoptosis in human HT-29 colonic cancer epithelial cells	Ogunwobi and Beales (2006)
Adiponectin protects mice against DSS-induced acute colitis	Nishihara et al. (2006)
Adiponectin-deficient mice had more severe chronic colitis upon DSS treatment	Obeid et al. (2017)
Systemic adiponectin administration induced pro-inflammatory cytokine production; adiponectin deficiency protected mice against DSS-induced colitis	Fayad et al. (2007)
Adiponectin and AdipoR1 overexpression exacerbate DSS-induced colitis and increased proinflammatory cytokine production in human colon epithelial cell lines	Peng et al. (2018)

Several studies characterized the relation between LDL and IBD using statistical analyses of existing databases. Koutroumpakis et al. reported that persistent dyslipidemia was associated with disease activity of patients with IBD (Koutroumpakis et al., 2016). A 7.3-year long nationwide population-based study in Korea with a total over 9 million subjects found that lower total cholesterol (TC) levels, lower serum LDL levels and lower serum high density lipoprotein (HDL) levels were associated with a higher incidence of CD, but not UC. On the other hand, lower serum triglyceride levels were associated with higher incidence of UC (Soh et al., 2020). Similarly, IBD patients exhibited lower levels of LDL and total cholesterol compared with healthy subjects with that finding being more significant in CD than UC patients (Agouridis et al., 2011). Consistently, in a more recent study, Tao et al. carried out a genome-wide association study using the data of IBD patients from the UK Biobank and found that there was an inverse relationship between the levels of HDL and the risk of CD, suggesting a possible link between HDL and IBD. In addition, treatments with lipid lowering agents targeting different key enzymes in lipid metabolism yielded a discrete effect: genetically proxied inhibition of proprotein convertase subtilisin/kexin type 9 increased the risk of IBD, UC and CD, while inhibition of cholesteryl ester transfer protein decreased the risk of CD (Tao et al., 2023). Outside of the aforementioned clinical database analyses, only a few studies explored the functional role of LDL in IBD pathology using experimental models. For instance, LDL receptor KO (LDLr-/-) mice are protected from DDS-induced colitis with increased accumulation of intestinal tolerogenic Tregs (Rodia et al., 2019). Whether the effect of LDL in IBD pathogenesis as discussed above is mediated by T-cad remains to be elucidated.

## T-cad inhibitory or restoring agents for therapeutic purpose

Unlike other members in the Cadherin superfamily, such as P-Cadherin or CDH17, of which the therapeutic agents are actively trialed in human studies (Harding et al., 2022; Morelli et al., 2025), there are no specific anti-T-cad antibodies, inhibitors, antagonists or agonists currently being tested in clinical trials. The therapeutics targeting T-cad remains primarily in the preclinical stage. This is because T-cad functions in a highly context-dependent manner, especially in different types of cancers. However, highly specialized monoclonal antibodies targeting T-cad have been engineered in preclinical studies. Recently developed Ca13Mab-17 (Shimizu et al., 2026), targeting both the mature form and uncleaved precursor of human T-cad, has been successfully used in preclinical laboratory models to identify glioblastoma cells and new tumor blood vessels, showing promising outcomes for future targeted diagnostics and antibody-based therapies. Since there is no direct small-molecule antagonist for T-cad available, agents aiming at indirect inhibition or downstream modifications are being developed. As the agonist to Liver X Receptors (LXRs) which are special intracellular proteins that act as master sensors for cholesterol and fat, the oral administration of GW3965 was shown to successfully counter T-cad-mediated vascular dysfunction and alleviate pathological neovascularization in preclinical models of neovascular age-related macular degeneration (Yin et al., 2026). Bioinformatics and *in vitro* screening models have predicted that multi-kinase inhibitors including Foretinib and Elesclomol can significantly downregulate the expression of *CDH13* and *CDH2* and exert strong inhibitory effects on adrenocortical carcinoma cells (Situ et al., 2024). An interesting study (Staebler et al., 2024) showed that T-cad-expressing melanoma cells are highly sensitive to proliferation suppression and apoptosis induction by Garcinol, a natural compound isolated from the plant know as mangosteen (*Garcinia mangostana*). Inhibition of T-cad decreased the cellular sensitivity to Garcinol and restored proliferation and survival of the melanoma cells, positioning Garcinol as a promising natural compound targeting at T-cad. The *CDH13* gene promoter is significantly hypermethylated in patients with UC (Tahara et al., 2014), which results in the downregulated gene expression of T-cad. Therefore, the therapeutic strategy targeting T-cad in IBD could be to restore its expression and function instead of inhibition, especially given its established role in mitigating EMT and suppressing adipogenesis. Intestinal fibrosis from IBD often becomes self-propagating - the intestinal wall structure, tissue remodeling and stiffness continue to progress even after the complete clearing of mucosal inflammation (Wang et al., 2022; Rieder et al., 2024). Therefore, for CD patients who have already progressed to the fibrostenotic phase, modulating T-cad might only offer quite a limited therapeutic benefit given that its application window is most likely limited to very early, preventative intervention. In addition, since T-cad is highly expressed in the nervous system, cardiovascular and epithelium of various types of tissue, any therapeutic approach, either trying to rescue expression or selectively blockage, may trigger significant systemic side effects.

## Summary and perspective

This review provides evidence that T-cad plays crucial and diverse roles in regulating the activity of various cell types, maintaining normal functionality and homeostasis across multiple tissues and organs. Consequently, aberrant T-cad expression and function is linked to diverse pathologies including neurological and cognitive disorders, cardiovascular and metabolic diseases and malignancies. Mechanistically, T-cad drives these processes through homophilic intercellular adhesion or by serving as a surface receptor for LDL and HMW-adiponectin. Despite recent insights into T-cad biology, further gain- and loss-of-function investigations remain essential to fully elucidate its tissue-specific ligand binding and downstream signaling pathways. Moreover, there is urgent need to define its precise impact on disease pathologies, particularly within the context of IBD, to facilitate clinical translational application. Given its established functions in other organ systems as discussed in this review, T-cad quite likely plays a key role in intestinal inflammation and stricture development. Ultimately, prospective studies will help us better understand the role of T-cadherin in IBD and its associated comorbidities, including the intestinal fibrosis.
